# Improving Pain Self-Management Among Rural Older Adults With Cancer

**DOI:** 10.1001/jamanetworkopen.2024.21298

**Published:** 2024-07-17

**Authors:** Megan J. Shen, Tammy Stokes, Sarah Yarborough, Jill Harrison

**Affiliations:** 1 Division of Clinical Research, Fred Hutchinson Cancer Center, Seattle, Washington; 2 Maury Regional Medical Center, Columbia, Tennessee; 3 Department of Health Services, Policy, and Practice, School of Public Health, Brown University, Providence, Rhode Island

## Abstract

**Importance:**

Undertreated cancer pain is a major public health concern among older adults in rural communities. Interventions to improve pain management among this vulnerable population are needed.

**Objective:**

To test the feasibility, acceptability, and changes in pain outcomes from exposure to an adapted intervention, Cancer Health Empowerment for Living without Pain (CA-HELP), to improve patients’ communication about pain to their clinicians.

**Design, Setting, and Participants:**

Older adults with cancer (aged ≥65 years) who were residing in a noninstitutional rural setting and receiving outpatient care at a rural-based clinic in Tennessee were enrolled in the study, in which everyone received the intervention, in May 2022. All patients were given assessments at baseline and 1 week after intervention. Mean score differences were analyzed using 1-tailed paired sample *t* tests (α = .05). Data were analyzed in June 2022.

**Exposure:**

The adapted version of CA-HELP included an 18-page patient-facing workbook and a 30-minute telephone coaching call with a registered nurse to coach patients on pain education and communication techniques to discuss pain with their medical team.

**Main Outcomes and Measures:**

Feasibility was examined through accrual and completion rates. Acceptability was measured by helpfulness, difficulty, and satisfaction with the intervention. Changes in outcomes were measured using mean score differences from pre-post assessments of pain self-management, self-efficacy for communicating with clinicians about pain, patient-reported pain, and misconceptions about pain.

**Results:**

Among the 30 total participants, the mean (SD) age was 73.0 (5.1) years; 17 participants (56.7%) were female, 5 (16.7%) were Black or African American, 30 (100%) were non-Hispanic or non-Latino, 24 (80.0%) were White, 16 (53.3%) had less than a high school education, and 15 (50.0%) reported income less than $21 000 per year. Based on accrual and completion rates of 100%, this intervention was highly feasible. Fidelity rates for delivering intervention components (100%) and communication competence (27 participants [90%]) were also high. Regarding acceptability, all patients rated the intervention as helpful, with the majority (24 participants [80%]) rating it as “very helpful.” Most patients rated the intervention as “not at all difficult” (27 participants [90%]), enjoyed participating (21 participants [70%]), and reported being “very satisfied” (25 participants [83.3%]). Pre-post changes in outcomes suggested significant improvements in pain self-management and self-efficacy for communicating with clinicians about pain, as well as significant reductions in patient-reported pain and pain misconceptions.

**Conclusions and Relevance:**

In this case-series study of CA-HELP, results suggested the adapted version of CA-HELP was feasible and acceptable and showed changes in pain-related outcome measures among older adults with cancer in a rural setting.

## Key Points

**Question:**

Is the adapted version of Cancer Health Empowerment for Living without Pain (CA-HELP) feasible, acceptable, and able to improve pain outcomes among older adults with cancer living in rural settings?

**Findings:**

Study findings from this case series design study of 30 patients suggested the adapted version of CA-HELP was feasible, acceptable, and able to improve patients’ communication about their pain to their clinicians.

**Meaning:**

Study results highlight a potentially low-cost, low-burden intervention designed to improve pain communication and reduce pain severity and pain misconceptions among older adults with cancer in rural settings.

## Teaser

This case series study evaluates feasibility, acceptability, and changes in pain outcomes from exposure to an adapted intervention to help patients with cancer communicate with their clinicians about pain.

## Introduction

The burden of pain in people living with cancer is well documented,^1,2^ and effective pain management is one of the largest population health challenges among older adults in the US.^3,4,5,6,7,8,9,10^ It is estimated that as many as 80% of people diagnosed with cancer experience pain.^11^ Among the older adult population living in the rural US, undertreated cancer pain is especially common.^12,13^ Older adults living in rural areas are disproportionately affected due to concerns about the overuse of pain medication,^5^ less access to care,^6^ preferences for nonpharmacological interventions,^8^ and reluctance to talk about pain.^14^

Older adults with cancer are commonly afraid to voice pain concerns as they believe it reflects worsening cancer, is just part of life, and do not want to burden others.^1,12,13,15,16^ As such, older adults may experience greater pain burden than the general patient population with cancer due to failing to bring up pain management as a concern to their clinicians. Further burdening older adults in rural settings is the reality that communication about pain in these settings exists against the backdrop of a growing opioid crisis that disproportionately impacts these communities.^17,18^ This backdrop can impact patients’ willingness to seek out pain management and clinicians’ need for considering nonpharmacological treatment options to manage pain. There is an urgent need for interventions targeting pain management communication between older adults with cancer in rural communities and their clinicians; however, this need outpaces the current evidence base.

To address this need, we conducted a National Institutes of Health (NIH)–funded pilot study to adapt and test a promising evidence-based intervention, Cancer Health Empowerment for Living without Pain (CA-HELP). Prior research indicates CA-HELP demonstrated significant improvement among patients with cancer in their self-efficacy to communicate with their physicians about their pain.^19,20^ Grounded in social-cognitive theory,^21,22^ which posits that behavior change and maintenance depends largely on individuals’ ability and self-efficacy to execute a specific behavior, CA-HELP coaches patients to ask questions, make requests, and signal distress to their clinicians to achieve improved cancer pain control. Although a promising tool among older adults with cancer, the original CA-HELP intervention was not designed for optimal implementation in rural settings or among older adults.

### Adaptation of Ca-HELP to Serve Older Adults With Cancer in Rural Settings

We adapted the CA-HELP (CA-HELP-A)^23^ to the older adult patient population with cancer in rural settings using the Method for Program Adaptation through Community Engagement (M-PACE)^24,25^ model by partnering with older adult patients with cancer, their informal caregivers, and clinicians. Based on their feedback, modifications were made to the original intervention using the Framework for Reporting Adaptations and Modifications-Enhanced^26^ model. The adapted version was tailored to meet the needs of older adults in rural settings, which is outlined in the original article.^23^

The goal of the present study was to pilot test the adapted version of the intervention, which included a patient workbook and 30-minute telephone call with a nurse interventionist, in a busy rural cancer clinic with older adults with cancer. Based on prior work, our hypotheses were that the CA-HELP-A intervention would be highly feasible and acceptable. Although the focus of this study was to examine feasibility and acceptability, we also explored if there were significant improvements in pain self-management and self-efficacy to communicate about pain with clinicians as well as reductions in patient-reported pain and pain misconceptions.

## Methods

### Participants and Procedures

All study procedures were approved by the institutional review board of Maury Regional Medical Center, Columbia, Tennessee. All participants provided informed consent and were recruited in May 2022 from an outpatient oncology clinic in rural Tennessee. Study staff conducted medical record reviews through the electronic medical record (EMR) to identify eligible patients. Eligibility criteria for patients included (1) receiving a diagnosis of cancer, (2) being aged 65 years or older, (3) speaking English, (4) residing in a noninstitutional rural setting, (5) receiving care at a community-based rural clinic, and (6) being able to provide informed consent. Exclusion criteria included (1) severe cognitive impairment (as indicated by medical team members) and (2) receiving hospice at the time of enrollment. Based on these eligibility criteria, patients were able to receive palliative care consultations or treatment during the course of the study. Study participants consented verbally via telephone or in person. This was an open group study; thus, all study participants received the study intervention. This study followed the reporting guideline for case series.

After consenting to the study, participants were given a baseline questionnaire assessing demographics, clinical characteristics, and targeted primary outcome (pain self-management) and secondary outcomes (self-efficacy for communicating with physicians about pain, pain misconceptions, and patient-reported pain). Next, participants were given a printed copy of the patient intervention (CA-HELP-A) workbook either in person or via mail. After receiving this workbook, participants engaged in a 30-minute intervention session over the telephone with a study staff nurse focused on reviewing modules and exercises included in the workbook. The nurse was a clinical lead in the palliative care clinic and thus was knowledgeable about pain management. Finally, after completing the intervention session, participants completed a postintervention assessment that included primary and secondary outcomes as well as measures of acceptability. Assessments were conducted in person or via the telephone by study staff. Because the primary goal of the study was to test the feasibility and acceptability of the intervention, the proposed sample size was 30. This number was selected based on recommendations in the Obesity-Related Behavioral Intervention Trials (ORBIT) model of research for developing and testing behavioral interventions.^27^ This study set out to complete phase IIa (proof-of-concept) of the ORBIT model, in which the study goal is to focus on delivering a treatment-only design.^27^ The ORBIT model deems sample size calculations to be unnecessary during this phase since the focus is on testing the feasibility of delivering the intervention.

### Adapted Intervention: CA-HELP-A

The original CA-HELP is an evidence-based communication tool that empowers and engages patients to communicate effectively with their physicians about pain.^17,18^ This intervention consists of 6 modules: (1) assessment of current knowledge, attitudes, and preferences around pain control; (2) clarification and correction of misconceptions about cancer pain control; (3) teaching of relevant concepts (education about cancer pain control); (4) planning (identifying pain goals, creating achievable pain management goals, and creating strategies to communicate these goals to clinicians); (5) rehearsal of communication strategies using role play exercises; and (6) portrayal of learned skills (patient applies skills in visit with health care clinician). We adapted this original version to meet the needs of older adults with cancer receiving care in rural settings.^23^ Major modifications from the original CA-HELP intervention to the current version (CA-HELP-A) included adding a patient-facing workbook to guide the intervention session and inclusion of visual imagery, reduced number of words and complexity to meet a sixth grade reading level, stigma around pain management, and acknowledgment that pain should not be normalized due to age or disease status. The adapted version of the intervention was pilot tested in the present study.

This adapted version (CA-HELP-A) resulted in an 18-page patient-facing workbook, of which 9 pages include active materials (eg, learning content or exercises). The workbook consisted of 5 modules, instead of 6, which were collectively retitled “5 Steps for Talking with Your Doctor about Pain When Living with Cancer” with each module renamed to “Steps” based on patient and informal caregiver feedback. The 5 steps include step 1: checking awareness (action), step 2: clarifying (correct), step 3: gaining knowledge (learn), step 4: setting goals (plan), and step 5: talking to your doctor (practice). The final version of the patient workbook was composed largely of image-based communication, including pain scale ratings based on imagery and color, assessments based on worries (thumbs up or down ratings), and exercises to determine pain management approaches patients would like to have and setting goals and strategies for talking to their clinician.

### Measures

#### Demographic, Disease, and Clinical Characteristics

Patients self-reported their age (in years), sex (male/female), ethnicity (Hispanic/Latino vs non-Hispanic/non-Latino), race (American Indian or Alaskan Native, Asian, Black or African American, Native Hawaiian or other Pacific Islander, White, multiracial, other [Hispanic or Latino/Latina], or other), relationship status, employment status, highest education level completed, total household income, insurance status (insured vs not insured), and primary treatment payment type. Race and ethnicity were assessed in this study to clearly outline sociodemographics of the patient population served and recruited. Disease characteristics were extracted from the EMR, including comorbidity status, cancer type and site, current cancer stage, presence of metastasis (yes vs no), and pain medication usage. Performance status was also extracted from the medical record and assessed with the Eastern Cooperative Oncology Group (ECOG)^28,29^ and/or Karnofsky performance status.^30^

#### Feasibility, Acceptability, and Treatment Fidelity

Intervention feasibility was assessed with accrual and intervention and study procedure completion rates. Acceptability was assessed postintervention. Participants rated the overall perceived helpfulness of the intervention and satisfaction with the intervention. The helpfulness item was rated on a 5-point scale from 1, not at all helpful, to 5, very helpful (“Overall, how helpful was the intervention to you?”). The satisfaction item was rated on a 5-point scale from 1, not at all satisfied, to 5, very satisfied (“How satisfied were you with the intervention?”). Additionally, participants rated the intervention difficulty with a single item (“How difficult was it for you to understand the content of the intervention?”; 1, not at all, to 5, very much). Additional features of the intervention were rated including satisfaction with session length, satisfaction with amount of session information, intervention delivery modality (in person or phone), workbook modality (paper vs electronic or online version), and preference for inclusion of a caregiver.

Treatment fidelity was assessed with a checklist that captured whether: (1) the interventionist demonstrated communication consistency (eg, pacing, volume, and introduction to the purpose of the intervention), and (2) delivered core intervention components. These checklists were completed for all intervention sessions by trained fidelity raters who listened to audio recordings of the intervention sessions. Fidelity was defined as delivering 70% or more of intervention components and using 70% or more of the therapeutic techniques.

#### Pain Self-Management

Pain self-management, which is the targeted primary outcome for future trials, was measured using 2 items from the pain management subscale of the Chronic Pain Self-Efficacy scale.^20^ Items on this scale are rated on a 5-point Likert scale (1, not at all certain, to 5, extremely certain) in which patients rate the degree of certainty they have about their ability to manage their pain (eg, “How certain are you that you can decrease your pain quite a bit?”).

#### Self-Efficacy for Communicating With Physicians About Pain

Self-efficacy for communicating with physicians about pain was assessed using the 5-item Perceived Efficacy in Patient-Physician Interactions scale^31^ as modified to refer to communication with oncologists.^20^ Items are rated on a 5-point Likert scale (1, not at all confident, to 5, very confident) in which patients indicate how confident they are about communicating with their physician (eg, “How confident are you in your ability to know what questions to ask your cancer doctor?”).

#### Pain Misconceptions and Patient-Reported Pain

Pain misconceptions were assessed using 11 items based on the Short Form Barriers Questionnaire (SBQ).^32^ Items are rated on a 5-point Likert scale (1, disagree very much, to 5, agree very much) in which patients indicate the degree to which they agree with certain statements about pain (eg, “It doesn’t do any good to talk about pain”). Patient-reported pain was assessed as the mean of the average and worst pain over the past 2 weeks on a 0 to 10 scale (0, no pain to 10, worst pain imaginable).^20^

### Statistical Analysis

Descriptive statistics were used to examine demographic, disease, and clinical characteristics. Mean score differences were examined between preintervention and postintervention outcomes using 1-tailed paired sample *t* tests. Significance was set an α level of .05. Individual *t* tests were run for each of the following outcomes: pain self-management, self-efficacy to communicate about pain, pain misconceptions, and patient-reported pain. In regard to missing data, 1 participant was removed from the pain severity subscale analysis due to missing postseverity scores. Data were analyzed in December 2022 with R version 4.2.1 (R Project for Statistical Computing).

## Results

### Demographic, Disease, and Clinical Characteristics

A total of 30 patients were enrolled in the study and completed the intervention. They had a mean (SD) age of 73.0 (5.1) years; 17 (56.7%) were female, 5 (16.7%) were Black or African American, 30 (100%) were non-Hispanic or non-Latino, 24 (80.0%) were White, and 16 (53.3%) were married or partnered. Most patients had a high school education or less (16 patients [53.3%]) and reported being low income, with 15 (50.0%) reporting a household income of less than $21 000 and 14 (46.7%) reporting a household income of $21 000 to $39 000. In terms of disease and clinical characteristics, patients had a mean (SD) Karnofsky score of 8.0 (1.9) and ECOG score of 1.0 (1.0). The largest portion of patients were stage IV (12 patients [40.0%]) and most patients were not currently taking pain medications (21 patients [70.0%]). See the Table for all patient demographic, disease, and clinical characteristics.

**Table.** Demographic, Disease, and Clinical Characteristics for Patients

Characteristic	Participants, No. (%)
Age, mean (SD), y	73 (5.1)
Sex	
Male	13 (43.3)
Female	17 (56.7)
Hispanic/Latino	
Yes	0
No	30 (100.0)
Race	
Black or African American	5 (16.7)
White	24 (80.0)
Multiracial	1 (3.3)
Relationship status	
Married/partnered	16 (53.3)
Divorced	5 (16.7)
Separated	1 (3.3)
Never married	1 (3.3)
Widowed	7 (23.3)
Employment status	
Employed, full-time	2 (6.7)
Employed, part-time	1 (3.3)
Retired	27 (90.0)
Highest education level completed	
≤High school	16 (53.3)
Some college	6 (20.0)
≥College	8 (26.7)
Total annual household income	
≤$21 000	15 (50.0)
$21 000-$39 000	14 (46.7)
Refused	1 (3.3)
Insurance status	
Insured	30 (100.0)
Not insured	0
Primary treatment payment type^a^	
Medicaid	1 (3.3)
Medicare	27 (90.0)
Private health insurance	10 (33.3)
Health maintenance organization	2 (6.7)
Other government funded/subsidized coverage	0
Self-pay	0
Comorbidity status	
Comorbidity, present	8 (26.7)
Comorbidity, not present	22 (73.3)
Karnofsky score, mean (SD)	8 (1.9)
Eastern Cooperative Oncology Group score, mean (SD)	1 (1.0)
Cancer type/site	
Bladder	1 (3.3)
Breast	6 (20.0)
Colorectal	2 (6.7)
Gallbladder/biliary	1 (3.3)
Gynecologic	4 (13.3)
Head and neck	2 (6.7)
Kidney	1 (3.3)
Lung	4 (13.3)
Pancreatic	1 (3.3)
Prostate	3 (10.0)
Other	5 (16.7)
Current cancer stage	
I	7 (23.3)
II	4 (13.3)
III	4 (13.3)
IV	12 (40.0)
Not in medical record	3 (10.0)
Metastasis	
Yes, metastasis indicated	12 (40.0)
No, metastasis not indicated	18 (60.0)
Pain medication usage	
Currently taking medication(s) for pain	9 (30.0)
Not currently taking medication(s) for pain	21 (70.0)

^a^ Some individuals indicated multiple payment types, so total column percentage exceeds 100%.

### Intervention Feasibility

Feasibility was defined as 70% or more of eligible participants enrolling in the study, as well as 70% or more of enrolled participants completing the intervention. Study staff attempted to reach 39 patients for the study. Of those, 5 were deemed ineligible due to age or death, and 4 refused to participate. Thus, a total of 30 participants were eligible and consented. All 30 consented participants enrolled in the study and completed baseline assessments, the intervention session, and postintervention assessments. There were no patients lost to follow-up.

### Intervention Acceptability

Regarding acceptability, all 30 participants rated the intervention as helpful, with the majority (24) rating it as “very helpful” (mean [SD] score, 4.80 [0.50]). Overall, 25 participants (83.3%) reported being “very satisfied” with the intervention (mean [SD] score, 4.70 [0.64]), and 21 (70.0%) reported enjoying participating in the intervention. Most participants (28 patients [93.3%]) found 1 session to be the right length and found the intervention to contain the right amount of information. Most participants (27 patients [90.0%]) rated the content of the intervention as “not at all difficult” to understand (mean [SD] score, 1.3 [1.02]). The 3 participants who indicated some degree of difficulty did not specify the aspects they found difficult to understand.

A total of 26 participants (86.7%) indicated a preference for either an in-person or phone session with a health coach, nurse, or social worker. Almost all participants (29 patients [96.7%]) preferred receiving a paper version of the workbook over an electronic or online version. Finally, a total of 25 participants (83.3%) indicated they would have liked participating in the session with a caregiver or loved one.

### Treatment Fidelity

Fidelity was defined as using 70% or more of the communication competencies and delivering 70% or more of core intervention components. Fidelity rates of 90% for communication consistency (pacing, volume, and introduction to purpose of intervention) and 100% for delivery of intervention components were observed, meeting the benchmarks for treatment fidelity across both categories.

### Changes in Pain-Related Outcomes

A total of 30 patients were enrolled, adequately powering this study (0.85) to detect moderate effect size differences. Pre-post changes in outcomes suggested significant improvements in pain self-management and self-efficacy for communicating with physicians about pain, as well as significant reductions in patient-reported pain and pain misconceptions. Pain self-management scores saw a mean (SD) total score increase of 1.8 (1.45) points (*t*_29_ = 6.809; *P* < .001). Patient-reported pain scores had a mean (SD) score difference of 0.5 (1.57) points (*t*_28_ = 1.715; *P* = .049). Pain misconception scores had a mean (SD) score difference of 1.4 (0.43) points (*t*_29_ = 18.281; *P* < .001). Finally, scores for self-efficacy for communicating with physicians about pain had a mean (SD) score increase of 2.8 (2.86) points (*t*_29_ = 5.297; *P* < .001). See the Figure for a visualization of results from this pilot study.

**Figure.**
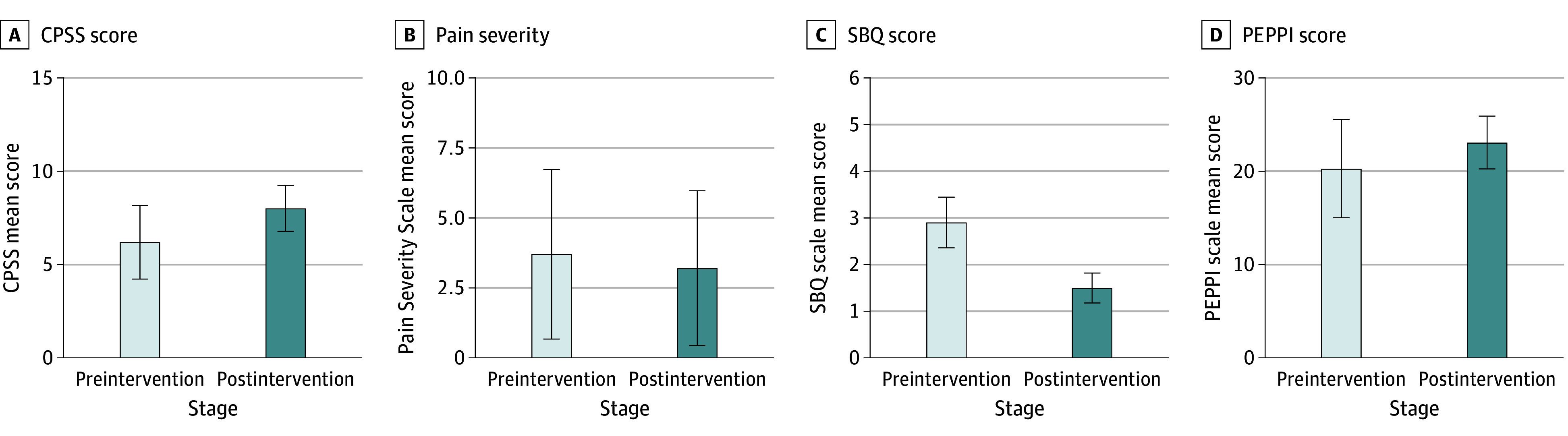
Change Scores in Outcomes From Preintervention to Postintervention CPSS indicates Chronic Pain Self-Efficacy scale (to measure pain self-management); PEPPI, Perceived Efficacy in Patient-Physician Interactions scale (to measure self-efficacy for communicating with physicians about patient-reported pain); SBQ, Shortened Barriers Questionnaire (to measure pain misconceptions). *P* values are as follows: A, *P* < .001; B, *P* < .05; C, *P* < .001; D, *P* < .001. Error bars indicate SDs.

## Discussion

In this case-series study among older adults with cancer in rural settings, we examined the feasibility, acceptability, fidelity, and pre-post results of a communication-based intervention designed to improve pain self-management and pain communication with clinicians among older adults with cancer receiving care in rural settings. Results suggested the adapted version of CA-HELP (CA-HELP-A) was feasible, acceptable, had high rates of intervention fidelity, and demonstrated potential efficacy at improving targeted pain outcomes. Rates of enrollment (88.2%) and completion of intervention sessions and study procedures (100.0%) were outstanding, demonstrating high rates of feasibility among this difficult-to-reach population. Patients also reported high rates of acceptability, including rating satisfaction with the intervention and helpfulness of the intervention highly and difficulty of the intervention low. Additionally, most participants reported enjoying participating in the intervention and preferring the chosen mode of intervention delivery (telephone) as well as workbook medium (printed, hard copy).

Most promising, study results suggest significant improvements in patients’ self-reported pain self-management and self-efficacy to communicate with physicians about pain as well as significant decreases in pain misconceptions and patient-reported pain. These results suggest that CA-HELP-A may be an effective intervention at improving multiple pain outcomes among older adults with cancer living and receiving care in rural settings. Given the high rates of undertreated cancer pain among older adults living in the rural US,^12,13^ the results of this pilot study are especially promising. Improving the capacity of rural clinics to identify older adults with cancer who are experiencing pain and are most likely to benefit from a pain management communication intervention is a high value proposition. These are heavily burdened clinics with very limited resources, often serving large catchment areas where patients must travel long distances to receive care.

Our adapted intervention (CA-HELP-A) is a low burden, light touch, fully remote, and potentially effective intervention for improving pain management among older adults in these settings. CA-HELP-A coaches patients in how to have conversations about their pain with their clinicians and develop an action plan to comanage it. As such, it is agnostic to pain intervention type. This allows patients to have honest conversations with their clinicians about their pain to find the best pain management approach for each patient. This holds promise for intervening on pain in a variety of ways, including using nonpharmacological approaches, which are the most commonly preferred methods of pain management among this vulnerable population.^8^ Additionally, this intervention could help address patients’ reluctance to talk about pain,^14^ overcome their misconceptions that pain is just a part of life,^1,12,13,15,16^ and bring up conversations about pain in a way that is destigmatizing amidst the growing opioid crisis affecting rural communities.^17,18^ CA-HELP-A is designed to target these barriers by encouraging patients to talk with their clinicians about their pain needs and to explore multiple options for pain management, including both opioids and nonpharmacological treatments. Future research should examine how the CA-HELP-A differs in potential efficacy among those taking pain medications vs those who are not. This may provide further insight on how to best target patients most in need of interventions to reduce their pain severity and improve their pain self-management. Additionally, future randomized clinical trials testing the efficacy of CA-HELP-A compared with a control condition should examine differences in how this intervention affects pain-related outcomes among patients based on disease severity (eg, early stage vs advanced metastatic cancer) and source of pain (eg, surgery, chemotherapy, radiation, or other chronic conditions).

### Limitations

Despite promising results, there are limitations that must be acknowledged in interpreting results of this study. First, although this sample was diverse in terms of education and income, it was less diverse racially and ethnically. The sample was predominately White and non-Hispanic/non-Latino. This is reflective of the study setting in rural Tennessee. Nevertheless, future research should examine the feasibility, acceptability, and potential efficacy of CA-HELP-A among more racially and ethnically diverse samples. Second, the CA-HELP-A intervention was only available and tested among English-speaking patients. Future work should expand this to include Spanish translations of the workbook to determine its potential utility among Spanish-speaking older adults in rural settings. Additionally, the sample size and pre-post design limit the ability to examine the efficacy of CA-HELP-A, control for key factors such as demographic variables that could impact treatment outcomes (eg, time, age, and disease severity), and generalize study findings. The main focus of the present study was on phase IIa (proof-of-concept) of the ORBIT guide for developing and testing behavioral interventions.^27^ In this phase, the goal of the study is to determine the feasibility of delivering the intervention and the intervention protocol, thus there is a lack of a control group to more stringently test the potential efficacy of the intervention relative to a control condition. Given this limitation, results around potential efficacy should be interpreted with caution. Future trials should examine the efficacy of CA-HELP-A among multiple sites with multiple patients.

## Conclusions

Preliminary evidence from this case-series study suggests that the CA-HELP-A intervention we adapted was highly feasible, acceptable, and significantly improved pain self-management and self-efficacy to communicate about pain with physicians and reduced patients’ self-reported pain and pain misconceptions. This holds great promise for addressing the urgent need to support the growing older adult population experiencing undertreated cancer pain.

## Notes

Supplement.
Data Sharing Statement
