# Supplementary material for: Improving Pain Self-Management Among Rural Older Adults With Cancer
Source: JAMA Netw Open. 2024 Jul 17;7(7):e2421298. doi: 10.1001/jamanetworkopen.2024.21298 (PMC11255907; doi:10.1001/jamanetworkopen.2024.21298)
Supplement: Supplement. — Data Sharing Statement [file jamanetwopen-e2421298-s001.pdf]

Data Sharing Statement

Shen. Improving Pain Self-Management Among Rural Older Adults With Cancer. *JAMA Netw Open*. Published July 11, 2024. doi:10.1001/jamanetworkopen.2024.21298

Data
Data available: No

Additional Information
Explanation for why data not available: Data will be shared upon request.
